# Reproducible Biofilm Cultivation of Chemostat-Grown *Escherichia coli* and Investigation of Bacterial Adhesion on Biomaterials Using a Non-Constant-Depth Film Fermenter

**DOI:** 10.1371/journal.pone.0084837

**Published:** 2014-01-03

**Authors:** Claudia Lüdecke, Klaus D. Jandt, Daniel Siegismund, Marian J. Kujau, Emerson Zang, Markus Rettenmayr, Jörg Bossert, Martin Roth

**Affiliations:** 1 Leibniz Institute for Natural Product Research and Infection Biology, Hans Knöll Institute (HKI), Bio Pilot Plant, Jena, Germany; 2 Faculty of Physics and Astronomy, Chair of Materials Science (CMS), Friedrich Schiller University Jena, Jena, Germany; 3 Otto Schott Institute of Materials Research (OSIM), Department of Metallic Materials, Friedrich Schiller University Jena, Jena, Germany; INRA Clermont-Ferrand Research Center, France

## Abstract

Biomaterials-associated infections are primarily initiated by the adhesion of microorganisms on the biomaterial surfaces and subsequent biofilm formation. Understanding the fundamental microbial adhesion mechanisms and biofilm development is crucial for developing strategies to prevent such infections. Suitable *in vitro* systems for biofilm cultivation and bacterial adhesion at controllable, constant and reproducible conditions are indispensable. This study aimed (i) to modify the previously described constant-depth film fermenter for the reproducible cultivation of biofilms at non-depth-restricted, constant and low shear conditions and (ii) to use this system to elucidate bacterial adhesion kinetics on different biomaterials, focusing on biomaterials surface nanoroughness and hydrophobicity. Chemostat-grown *Escherichia coli* were used for biofilm cultivation on titanium oxide and investigating bacterial adhesion over time on titanium oxide, poly(styrene), poly(tetrafluoroethylene) and glass. Using chemostat-grown microbial cells (single-species continuous culture) minimized variations between the biofilms cultivated during different experimental runs. Bacterial adhesion on biomaterials comprised an initial lag-phase I followed by a fast adhesion phase II and a phase of saturation III. With increasing biomaterials surface nanoroughness and increasing hydrophobicity, adhesion rates increased during phases I and II. The influence of materials surface hydrophobicity seemed to exceed that of nanoroughness during the lag-phase I, whereas it was *vice versa* during adhesion phase II. This study introduces the non-constant-depth film fermenter in combination with a chemostat culture to allow for a controlled approach to reproducibly cultivate biofilms and to investigate bacterial adhesion kinetics at constant and low shear conditions. The findings will support developing and adequate testing of biomaterials surface modifications eventually preventing biomaterial-associated infections.

## Introduction

Modern medicine increasingly uses biomaterials for implant purposes, e.g., to restore human body functions. Such implants can lead to infections by supporting adhesion of microorganisms and subsequent biofilm formation on the biomaterials surfaces [1], [2]. Understanding mechanisms of bacterial adhesion and biofilm formation is a crucial prerequisite to develop strategies for the prevention of biomaterial-associated infections (BAI). Suitable systems for biofilm cultivation and bacterial adhesion at controllable, constant and reproducible conditions are, therefore, indispensable [3]. Several *in vitro* model systems for biofilm cultivation and investigating microbial adhesion had been described and will be briefly summarized below for introducing the first aim of this study.

The most frequently used microtiter well plate systems allow for fast and high sample throughput and do not require specialized laboratory equipment [4]. Such systems are closed without in or outflow during the experiment [5], resulting in a change of culture conditions during the experiment, e.g. by accumulation of signaling molecules or metabolites, depletion of nutrients and oxygen. Many researchers use microbial cells that were washed, centrifuged and resuspended in buffer solutions to prevent growth during adhesion studies. These methods, however, lead to weakening or damaging the cells, which significantly affects bacterial adhesion [6]–[8], and altogether might preclude meaningful *in vitro* findings.

Different fluid displacement systems have been described, as well, for biofilm cultivation and microbial adhesion studies, respectively. These systems can be roughly divided into two groups [4]. The first includes systems where the fluid, i.e. bacterial culture, bacterial suspension or growth medium, is replaced unidirectional in the (axial) direction of flow. The fluid is mixed by diffusion in the direction of flow only. This group comprises, e.g. classical flow chambers [8]–[9], the modified Robbins device [10]–[11] and drip flow reactors [12]–[13]. Using these systems, bacterial adhesion and biofilm formation can be investigated under low and high shear conditions depending on the flow velocity. However, in these systems conditions change progressively and are not constant within the reactor. The second group includes fluid displacement systems where constant conditions within the reactor are achieved by perfect mixing of the fluid, e.g. with a stirrer. These systems comprise, e.g., the CDC (Centers for Disease Control) biofilm reactor [14] and the rotating disc reactor [15], and are used for investigating bacterial adhesion and biofilm formation at moderate to high shear conditions.

Infections related to biomaterials used in the human body can develop at low or high shear conditions depending on type and location of the medical device. Catheter-related infections of the urinary tract and the bloodstream are the most commonly occurring BAIs [16] developing mainly at moderate or high shear conditions with unidirectional fluid flow. Adequate *in vitro* model systems to investigate the biofilm formation at these conditions are available in form of different flow chamber devices. Infections related, for example, to bone fracture devices, joint replacements or cardiac pacemakers develop at low shear or nearly static conditions with slow multidirectional or no fluid flow. For investigating biofilm formation associated with these types of implants, model systems are needed which provide constant conditions in combination with low shear forces. To date, the only system almost meeting these requirements is the constant-depth film fermenter (CDFF) previously described by Peters and Wimpenny [17] and Kinniment *et al.* [18]–[19]. The CDFF is an established system extensively used for the investigation of oral biofilm (plaque) formation [4], [20]–[23]. Its main characteristic feature is the z (depth)-restriction of the cultivated biofilms by mechanical removal of excess biofilm with a scraper simulating the abrasive movement of the tongue [24]. Nevertheless, significant variations between the experimental runs have been observed, when microbial batch cultures, aliquots of saliva or chemostat-grown mixed populations of commensal oral bacteria were used for inoculation of the CDFF [19], [23]. Microbial cells grown in a chemostat (continuous culture) develop a *physiological steady state* with a constant cell density. This steady state is highly reproducible for single-species continuous cultures [25]. Thus, microbial cells grown in a single-species chemostat are a promising approach reducing the described variability and, furthermore, providing intact viable cells. The *first aim* of this study was, therefore, to modify the previously described CDFF system for the cultivation of non-z-restricted biofilms at constant and low shear conditions. Using the non-constant-depth (i.e., non-z-restricted) film fermenter (nCDFF), biofilms of *Escherichia coli* sourced from single-species continuous culture was cultivated on titanium dioxide (TiO_2_), one of the biomaterials used most for implant purposes [26], and tested for reproducibility.

When biomaterials are implanted in the human body, microbial colonization competes with tissue cell integration on the biomaterials surfaces [27]. Microbial adhesion is inhibited on implants covered by tissue cells and intact extracellular polymers [28]–[29]. Developing anti-microbial biomaterials surfaces to lower microbial adhesion rates would be crucial for preventing BAIs. Consequently, detailed and further understanding of adhesion kinetics *versus* biomaterials surface properties is essential. However, such studies most often do not seek correlation of microbial adhesion with time and materials surface structure and properties. Moreover, the types of biomaterials are diverse comprising polymers, ceramics and metallic materials. They substantially differ in their physical as well as chemical surface properties which, in turn, affect microbial colonization. For example, biomaterials surface nanoroughness [30]–[35] and surface hydrophobicity [8], [36]–[38] had previously been shown to significantly influence microbial adhesion. To evaluate the composite impact of these factors on microbial adhesion, multi-factorial investigations are necessary. The *second aim* of this study was, therefore, using the nCDFF to analyse bacterial adhesion over time on various relevant biomaterials, which comprise significantly different nanoroughnesses and cover a broad range of surface hydrophobicity. Adhesion of *E. coli* was investigated on TiO_2_, tissue culture poly(styrene) (TCPS), poly(tetrafluoroethylene) (PTFE, i.e. Teflon) and silicate glass to investigate if surface nanoroughness or hydrophobicity is most directly correlated with bacterial adhesion kinetics on the biomaterials.

In this study the CDFF was modified and used for cultivating non-z-restricted biofilms at constant and low shear conditions. The use of a single-species continuous culture for inoculation of the nCDFF was assessed to reduce variations between experimental runs compared to multi-species continuous culture. Using the nCDFF for investigating bacterial adhesion kinetics is highlighted. Detailed *E. coli* adhesion kinetics on various relevant biomaterials are provided in correlation with biomaterials surface nanoroughness and hydrophobicity. In that way, general conclusions concerning the bacterial adhesion on a broad range of different biomaterials can be drawn as a first step towards eventually reducing BAIs.

## Materials and Methods

### Model Organism

*Escherichia coli* EC081 was used for biofilm cultivation and investigating bacterial adhesion kinetics. EC081 was obtained by transforming *E. coli* RV308 (*lac*74-*gal*ISII:OP308*str*A) ( = ATCC 31608), a K-12 derivative, [39] with plasmid pMK3c2GFPuv, resulting in strong constitutive production of the green fluorescent protein (GFPuv) [40] by the cells for monitoring cell adhesion and biofilm formation by means of confocal laser scanning microscopy (CLSM). A detailed description of construction and structure of pMK3c2GFPuv is provided as **Text S1** and in **Fig. S1**.

### Experimental Setup

**Figure 1:**
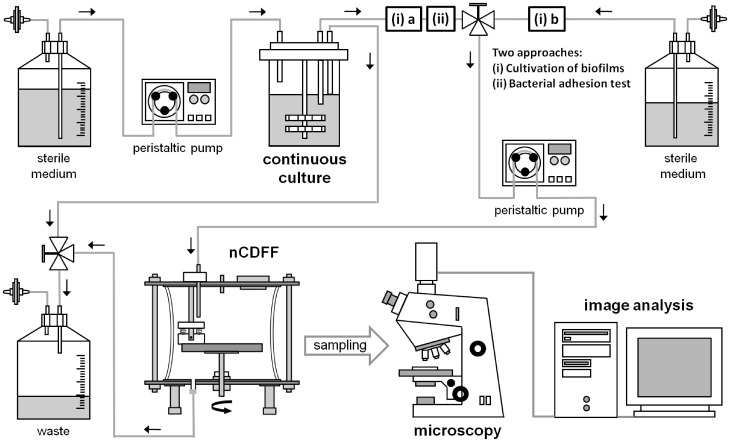
Overview of the experimental setup used in this study including continuous culture (chemostat), non-constant-depth film fermenter (nCDFF), microscopy and image analysis. Sterile culture medium is supplied via silicon tubes to the continuous culture (monitored by a control unit; not shown) using a peristaltic pump for adjusting the rate. Two approaches were used in this study for (**i**) the cultivation of biofilms and (**ii**) the test of bacterial adhesion. For biofilm cultivation, the nCDFF was (**a**) inoculated for a limited period of time and, afterwards, (**b**) sterile culture medium was supplied to enable biofilm growth. For adhesion tests, the nCDFF was inoculated throughout the experiment. The direction of flow is indicated using arrows. Bacterial samples were investigated using microscopy and subsequent image analysis.

An overview of the experimental setup and the two approaches used in this study are shown in **Fig. 1**. Silicon tubes connect the continuous culture (chemostat) monitored by a control unit (not shown) with a medium supply, a waste container and the nCDFF. Cultivation parameters such as temperature, pH or agitation and aeration are monitored and partly controlled. A peristaltic pump is used to continuously supply the nutrient medium to the culture vessel and for supplying the bacterial suspension or sterile growth medium, respectively, to the nCDFF.

For the cultivation of biofilms **(i)**, the materials samples within the nCDFF were inoculated with the continuous culture for a limited period of time **(a)** to allow bacterial adhesion. Subsequently, sterile culture medium was supplied to the nCDFF to enable biofilm growth **(b)**. For bacterial adhesion tests **(ii)**, the nCDFF was inoculated with the continuous culture throughout the experiment.

A flow chart additionally provided in **Fig. S2** visualizes the two approaches and depicts potential modifications of the experimental setup.

### Continuous Culture (Chemostat)

A continuous culture is defined by a specific microbial growth rate relative to its theoretical maximum controlled by the external substrate concentration of the limiting nutrient [25]. For continuous cultivation, a culture bioreactor was used with a certain flow rate (F). The culture volume (V) is maintained constant and the imposed dilution rate (D) is given by D = F/V. The theoretical generation time (T) of the microorganisms required for the constant density culture (steady state) in the fermenter is T = ln2/D. The specific growth rate µ of the microorganisms relative to its theoretical maximum is controlled by the concentration of a limiting nutrient, e.g. the carbon source or an inorganic ion, and is equal to the rate at which the culture is being diluted (µ = D).

*E. coli* was precultivated in 30 mL Luria Bertani broth in a 300 mL Erlenmeyer flask for 6 hours at 200 rpm and 30°C. Exponentially growing cells were harvested by centrifugation (2000 g, 10 min), washed twice in phosphate buffered saline (PBS) and resuspended in 80 mL PBS (10% of the continuous culture volume) for inoculation of the continuous culture. The optical density (OD_600_) of the continuous culture directly after inoculation was 0.18±0.05, which corresponds to ∼0.9×10^8^ cells/mL.

For the continuous culture a glucose-limited mineral medium containing (per liter) 4 g Na_2_HPO_4_⋅2 H_2_O, 3 g KH_2_PO_4_, 0.8 g MgSO_4_⋅7 H_2_O, 0.8 g glucose, 0.5 g NH_4_Cl, 0.5 g NaCl, 0.01 g FeCl_3_⋅6 H_2_O, 0.004 g MnCl_2_⋅4 H_2_O, 0.004 g thiamin, 0.002 g zinc acetate and 0.05 g kanamycin for plasmid maintenance was applied. Continuous culture was performed in a 1 L stirred bioreactor with 0.8 L working volume at 30°C and aeration with filter sterilized air (48 slpm). The culture was monitored using a digital control unit (DCU 3, B. Braun Biotech International, Melsungen, Germany), pH and oxygen concentration were monitored but not controlled. Cell growth was monitored by optical density measurements at 600 nm using a spectrophotometer (Spekol 1100, Analytic Jena, Germany). The waste reservoir was continuously monitored using a digital balance. After a batch period of 12–16 h at 30°C, the continuous culture was started with a flow rate F = 80 mL h^−1^ which gives a dilution rate D = 0.1 h^−1^ and a theoretical generation time T = 6.93 h. Steady state conditions were indicated by a constant OD_600nm_ of ∼0.55 (according to the defined dilution rate of the culture), a constant pH and oxygen concentration as well as glucose depletion. Inoculation of the biofilm reactor was started 1 to 3 days after reaching the steady state in the continuous culture.

### Non-constant-depth Film Fermenter (nCDFF)

A detailed description of the design and setup of the CDFF developed by Peters and Wimpenny [17] can be found elsewhere [24]. In brief, the CDFF consists of a glass vessel (Ø 18 cm, height 15 cm) and stainless-steel plates at the top and bottom. The top end owns a port for supplying either continuous culture broth or fresh medium, for aeration and for sampling. The bottom end plate provides an outlet for waste. The vessel houses a stainless steel disk (Ø 15 cm) with wells for sample deposition driven by a motor. The most characteristic feature of the CDFF is a scraper bar to restrict biofilm growth in height.

**Figure 2:**
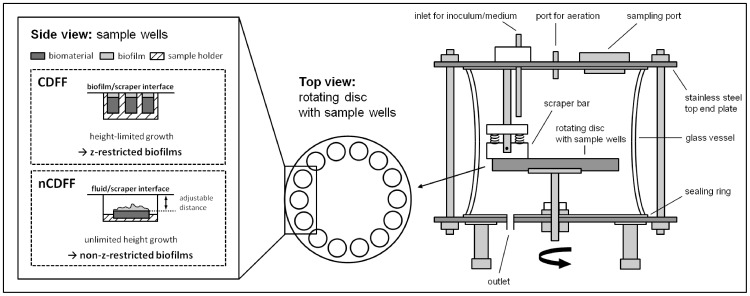
Illustration of the nCDFF and sample wells of the CDFF and nCDFF in comparison. Illustration (not to scale) of the non-constant-depth film fermenter on the right, consisting of a glass vessel and stainless steel end plates with ports for inoculation/sterile culture medium, aeration, sampling and an outlet. The vessel houses a rotating disc with 14 wells containing sample holders with the biomaterial samples. The bacterial suspension or medium, respectively, is continuously exchanged while the disc is slowly rotating. The scraper bar homogeneously distributes the inoculum/medium over the wells and, in addition, wipes off the surplus bacterial suspension/medium from the disc. On the left, the side view of a sample well including sample holder, biomaterial and cultivated biofilm of the CDFF [8] compared to the nCDFF (this study) is shown.

In this study, the CDFF was *modified* to cultivate biofilms without z-limitation at constant and low shear conditions **(** **Fig. 2** **)**. This was achieved by setting the distance between the scraper bar and the materials samples used for biofilm cultivation to a certain distance (adjustable) to prevent/avoid height limitation and disturbance of the growing biofilms by the scraper. In this study, the distance was set to constant 2 mm for biofilm cultivation and bacterial adhesion studies. Higher distances are recommended for studies intended for biofilm cultivation over longer periods of time. The scraper bar distributes the drops of incoming inoculum/medium over 14 round sample wells (Ø 23.5 mm) and wipes off excess bacterial suspension/medium. Thus, the scraper supports the continuous exchange of the inoculum/medium in the wells and encourages constant conditions for bacterial adhesion or biofilm growth in the wells. Furthermore, sample pans and holder were adapted for using varying types and sizes of biomaterials.

Continuous culture was supplied dropwise (drop volume ∼35 µl) at a constant rate of 60 ml per hour to the wells of the nCDFF to inoculate of the biomaterials samples. The drops were supplied to the wells near the walls (i.e. not directly above the materials samples). The sample well disc rotated at 2 rpm. Two drops were supplied to each well per minute (70 µL/min). Volume exchange of bacterial culture/medium in each well takes approximately 12.5 minutes (assuming perfect mixing) given the total liquid volume is 867.5 µL per well based on width of the liquid body (Ø 23.5 mm) in one well (corresponding to the diameter of the well) and height of the liquid above the sample holders (2 mm). The exchange time naturally depends on the volume of the liquid in each well and on the rate at which the liquid is exchanged.

The rate of supplying bacterial suspension or growth medium, respectively, is one factor affecting the shear conditions within each well. Low shear conditions are defined as conditions where fluid flow and mixing occurs with low flow velocities and, accordingly, low shear rates. The shear conditions within the sample wells of the nCDFF are determined by the drops of bacterial suspension or sterile medium to the wells and the movement of the scraper bar, respectively.

The shear rates occurring on the surface of the materials samples within the wells caused by these two factors were roughly estimated. Assuming for simplification a Hagen-Poiseuille flow, a shear rate of approximately 1.5 s^−1^ caused by the drops on the surface of the material sample was calculated based on a permanent average fluid flow of 1.19 µL/s and a height of the fluid body of 2 mm. This shear rate is assumed as the minimum value of the actual shear rate caused by each drop. Based on the assumption of a dropwise addition of fluid to each nCDFF sample well with a drop volume of 35 µL, a shear rate of 44.6 s^−1^ was applied two times per minute to the surface of the material sample. Nevertheless, the calculation is based on a diameter of the fluid body of 2 mm. Since the width of fluid body in the wells is higher (diameter of the well of 23.5 mm), this calculated value represents a maximum value, whereas the actual shear rate is lower.

The calculated shear rate at the surface of the material sample caused by the movement of the scraper bars (velocity of 12.6 mm/s) was approximately 6.3 s^−1^, assuming a fluid flowing between two parallel plates, of which one is moving at a constant speed and the other one remains stationary (Couette flow). These low shear conditions have to be distinguished from high shear conditions occurring in the medical praxis, e.g., in the lumen of catheters and stents. These conditions are commonly simulated in flow chambers used for the investigation of bacterial adhesion and biofilm formation.

The cultivation of the biofilms was carried out at a room temperature of 22.9±0.8°C (monitored with a data logger over 4 weeks). Glucose concentration in filtered samples from the continuous culture and the biofilm reactor was measured using an YSI 7100MBS bioanalyzer (Kreienbaum, Langenfeld, Germany) with a minimum detectable glucose concentration of 0.05 g/L.

### Preparation of Biomaterials

*E. coli* biofilms were cultivated on TiO_2_. TiO_2_ samples were prepared by physical vapor deposition as described previously [31]. In brief, glass slides (Ø 15 mm, thickness 0.7 mm; Borofloat® B33; Jena 4H Engineering GmbH, Jena, Germany) were used for titanium thin film deposition. Titanium was evaporated with an electron beam evaporator (Univex 350, Leybold, Germany) at a deposition rate of 0.5 nm/s to a film thickness of 200 nm at a vacuum of 6×10^−6^ Torr. The deposition rate was kept constant and controlled by a deposition monitor with an error range of 0.01 nm/s. Due to the contact of the evaporated titanium thin films with atmospheric oxygen, a titanium dioxide layer with an approximate thickness of 5 nm is immediately formed on the surface [41]. Samples were sterilized in the autoclave for 20 min at 121°C before use.

Bacterial adhesion kinetics on TiO_2_, TCPS, PTFE and silicate glass surfaces were investigated. Titanium samples were prepared as described above. TCPS discs with Ø 15 mm were produced from petri dishes (VWR Intenational GmbH, Darmstadt, Germany). A PTFE rod (purchased from Kahmann & Ellerbrock GmbH & Co. KG, Bielefeld, Germany) with Ø 15 mm was cut into 2 mm thick slices and ground with silicon carbide paper (Buehler GmbH, Düsseldorf, Germany) with decreasing grit sizes (P320, P600, P1200, P2500 and P4000). Glass slides were purchased from Jena 4 H Engineering GmbH (Borofloat® B33; Jena, Germany). TiO_2_ samples, PTFE slices and glass were heat sterilized (20 min at 121°C) before use in the nCDFF. TCPS samples were sterilized for 30 min in ethanol (70%) and afterwards rinsed with sterile distilled water.

### Characterization of Biomaterials

An atomic force microscope (AFM; Dimension 3100, Digital Instruments, Santa Barbara, CA, USA), equipped with a standard silicon tip was used to characterize the surface topography of the different biomaterials. AFM images were taken at three randomly chosen samples of each materials (n = 3) before use in the nCDFF. The AFM operated in tapping mode with a scan rate of 2 Hz. Images were acquired with a scan size of 1 µm ×1 µm and 5 µm×5 µm, respectively, and an image resolution of 512×512 points. The biomaterials root mean square surface roughness Rq and skewness Rsk were calculated with Gwyddion 2.28 free SPM data analysis software [42] according to equation (1) and (2) (ISO 4287–1997):
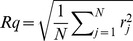
(1)

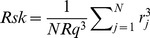
(2)


where N is the number of points (intersections of the roughness surface profile with the mean line), r_j_ the height of the profile at point j. The specific surface area of the biomaterials and the maximum vertical peak-to-valley distances were calculated based on the 1 µm×1 µm AFM images. For TiO_2_ and TCPS, furthermore, the number of peaks and the average distances between the peaks of surface line profiles of 1 µm length were estimated (n = 3 with 5 line profiles on each sample) to more specifically characterize the spatial distribution of the surface features.

The contact angles were obtained by sessile drop (static) method using distilled water drops (10 µL) (OCA20 drop shape analysis system, Data Physics, Filderstadt, Germany) on three samples of each biomaterial. Images of the sessile drops on the materials surfaces were recorded by a camera and analyzed using the software supplied by the manufacturer according to the tangent method.

### Biofilm Cultivation on Titanium Oxide

For *E. coli* biofilm cultivation (according to approach (i), **Fig. 1**), TiO_2_ samples in the nCDFF were inoculated for 21 hours (corresponding to about three doubling times of *E. coli* in glucose-limited mineral medium) to allow bacterial adhesion. To induce and maintain biofilm growth after inoculation, sterile culture medium with twice the glucose concentration (1.6 g/L) was supplied at identical flow rates as during inoculation. Four samples of each material with the cultivated biofilms were taken out of the nCDFF after 0.5, 1, 2, 3, 6 and 9 days. 3 samples were analyzed using CLSM (5 different points analyzed on each sample). One sample was used for scanning electron microscopy (SEM) imaging. The entire experiment was repeated three times to test for reproducibility of the cultivated biofilms.

### Bacterial Adhesion on Different Biomaterials

For investigation of bacterial adhesion over time (according to approach (ii), **Fig. 1**), biomaterials samples in the nCDFF were inoculated constantly with the continuous *E. coli* culture. Biomaterials with adhered bacteria were sampled at different time points. The optimal timing for sampling was roughly estimated in a pilot experiment. Using a microtiter well plate, samples of each material were incubated at room temperature for 6, 12 and 24 h with a batch culture of *E. coli* EC081 with an initial optical density measured at a wavelength of 600 nm of 0.5 to mimic the conditions in the nCDFF, and analyzed with CLSM (data not shown). Accordingly the points for sampling were set to: 1, 3, 6, 9, 12, 18 and 24 hours for TiO_2_, 1, 2, 3, 5, 7, 10 and 13 hours for TCPS and PTFE and 2, 6, 10, 16, 22, 30 and 38 hours for glass. Materials samples with adhered bacteria were analyzed using CLSM. The adhesion experiment was run once with n = 3 samples per time point and per material taken for analysis. To obtain a representative result for each sample, 5 randomly chosen points were analyzed each, giving an overall 15 different locations of analysis per time point and per material.

### Bacteria Sample Preparation for Microscopy

Samples for CLSM imaging and analysis were fixed with phosphate-buffered formaldehyde solution (4%, Roti®-Histofix, Carl Roth GmbH, Karlsruhe, Germany) for 12 h at 4°C and afterwards air-dried at room temperature in the dark. During preparation, samples were handled with care to prevent detachment and loss of adhered cells and biofilm, e.g. due to shear forces during rinsing. Dried samples were mounted on microscope slides, embedded using the ProLong® Antifade Kit (P7481, Molecular Probes®, Darmstadt, Germany) according to the manufacturer’s instructions and sealed with cover slips to prevent photo-bleaching effects during fluorescence microscopy and to enable long-time storage of the samples at −20°C. For SEM imaging, biofilm samples were fixed with glutardialdehyde solution (2.5% in PBS, pH 7.0) at room temperature for 12 h. After fixation, samples were carefully washed for 10 min in PBS buffer solution and twice in distilled water. Subsequently, samples were dehydrated using an ascending ethanol series from 10 to >99.9% with 10 min incubation for each step and two times 30 min for the last step. The dehydrated bacterial samples were critical-point dried (EMITECH K850, Quorum Technologies Ltd, East Grinstead, UK) and gold sputter coated (∼ 5 nm) (S150B, Edwards Ltd, Crawley, UK).

### Microscopy and Image Analysis

A confocal laser scanning microscope (Zeiss LSM 510 Meta, Carl Zeiss MicroImaging, Jena, Germany) equipped with an Argon laser (488 nm) and a 63× NA 1.3 oil immersion lens objective (Zeiss PLANAPOCHROMAT®) was used for fluorescence imaging of the bacteria. With a 1.5-fold digital zoom, the basic field of view for each image was 71.4 µm×71.4 µm. For analysis of the cultivated biofilms (approach (i)) on the titanium surfaces, image stacks were taken at five different randomly chosen points on each sample (n = 3 samples per time point and for each material). The approximate distance between two consecutive images within a stack was 0.5 µm resulting in 10 to 100 images for each stack depending on the thickness of the biofilm. The image stacks were analyzed using the free software bioImage_L v.2.1 [43]. For image analysis, a factor of 0.05 for noise reduction was used. Biofilm height and coverage were calculated for all biofilm samples from 0.5 to 9 days and the specific biofilm volume was calculated for samples taken at 3 to 9 days. Samples obtained during the bacterial adhesion experiment (approach (ii)) were characterized by calculating the material surface coverage based on five single CLSM images per sample with n = 3 samples per time point, and for each material using the same imaging parameters as described above.

For scanning electron microscopy imaging, an AURIGA 60 CrossBeam® FIB-SEM scanning electron microscope (Carl Zeiss AG, Oberkochen, Germany) was used at a magnification of 1000× to 10000× operated at 5 kV and a working distance of approximately 5 mm.

### Propagation of Uncertainty and Statistical Analysis

CLSM imaging of the biofilms and adhered bacteria, respectively, on the materials surfaces were carried out at five different randomly chosen points on each sample with n = 3 bacterial samples, thus, giving a total of 15 different locations of analysis per time point and for each material. Since the required quantity for statistical analysis was not obtained directly, for all analytical/mathematical operations which were performed on the measured quantities, a propagation of uncertainty was applied according to Taylor *et al.* [44]. For statistical analysis (one-way ANOVA) of the results, Statgraphics Centurion XV software (StatPoint Inc., Warrenton, USA) was used.

## Results

### Cultivation of *Escherichia coli*

**Figure 3:**
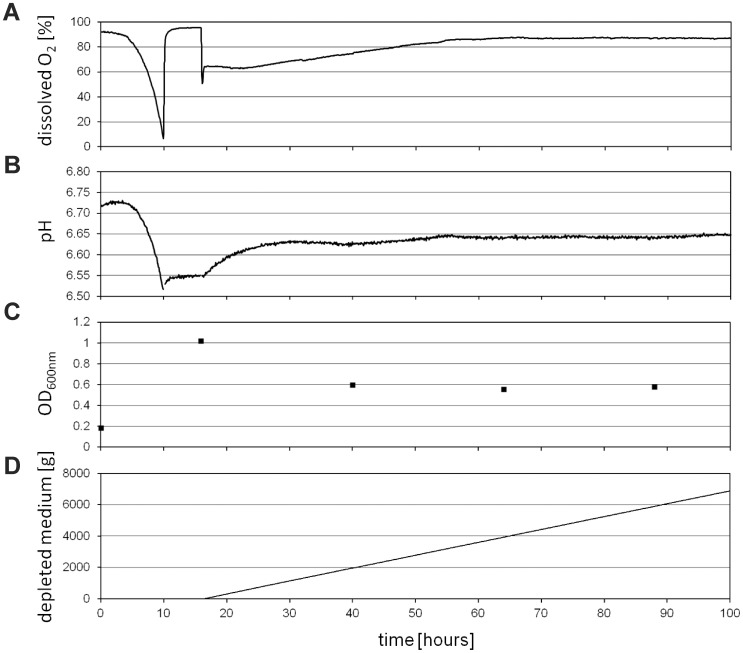
Culture parameters of the continuous culture of *E. coli*. Depicted are dissolved oxygen concentration (**A**), pH (**B**) and optical density (**C**) during the initial stage of the continuous cultivation of the model organism *E. coli*, including the batch phase after inoculation and the continuous phase indicated by the increasing weight (**D**) of the depleted medium from the culture. Oxygen concentration and pH in the culture were monitored but not controlled. The dilution rate (D) of the culture was 0.1 h^−1^. Doubling time of the bacteria was approximately 6.9 hours.

Dissolved oxygen concentration **(A)**, the pH **(B)** and optical density OD_600nm_ **(C)** for the initial 100 hours of the cultivation of *E. coli* including batch phase and continuous phase are shown in **Fig. 3**. In addition the weight of the collected waste is shown **(** **Fig. 3 D** **)** for monitoring the dilution rate (D) of the continuous culture. After 10 hours of cultivation in batch mode without continuous flow of medium through the culture vessel, the oxygen concentration and pH decreased from approximately 92.2% to 6.5% and from 6.7 to 6.5, respectively. This indicates the point of complete depletion of glucose within the culture and a halt in growth, since thereafter the oxygen concentration rapidly increased to 95.6%. With starting the continuous phase, the oxygen concentration rapidly decreased and reached a nearly constant level of 87% after 70 h of cultivation, while the pH increased to a value of 6.65. The optical density of the culture decreased from 1.0 at the end of the batch phase to 0.6 after 40 h which corresponds to approximately 4.7×10^8^ cells per mL, to remain nearly constant afterwards. The continuous culture reached a steady state after approximately 72 h of cultivation. At steady state conditions, the glucose concentration in the continuous culture broth was below the detection limit of 0.05 g/L.

### Biomaterials Surface Characterization

**Figure 4:**
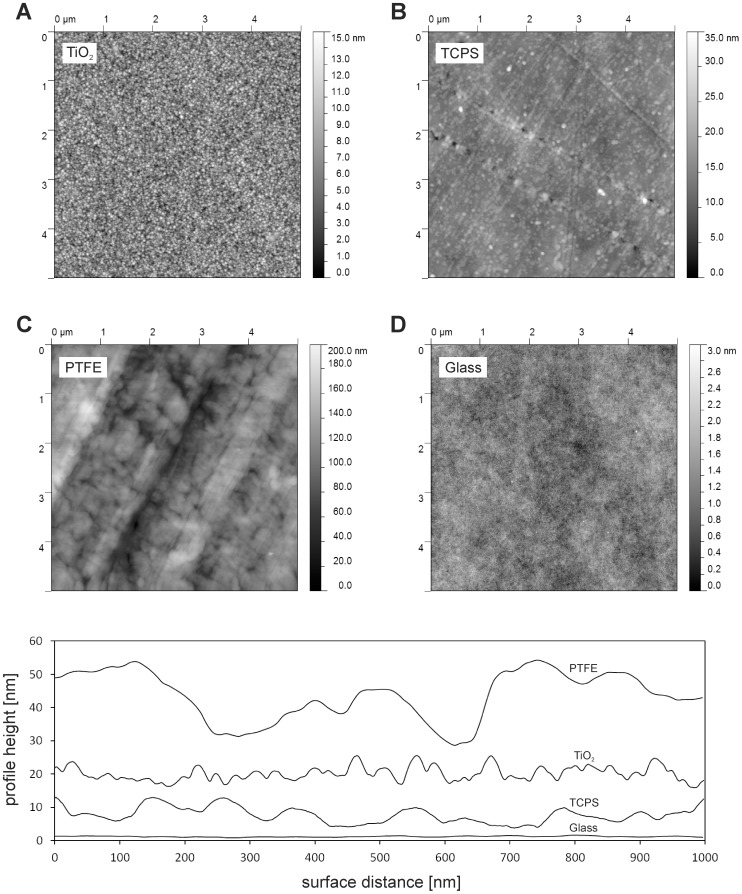
AFM height images and representative surface profiles of the biomaterials. AFM height images of the materials used in this study for biofilm cultivation and for investigation of bacterial adhesion: physical vapor deposited titanium thin films (**A**), tissue culture poly(styrene) (**B**)**,** poly(tetrafluoroethylene) (**C**) and Borofloat® silicate glass (**D**); AFM images were obtained in tapping mode in air, with a scan size of 5 µm×5 µm and a resolution of 512×512 pixel. The height profiles are exaggerated and set in offset (PTFE +40 nm, TiO_2_+20 nm, TCPS +8 nm, Glass +1 nm) for better visualization.

AFM height images (scan size 5 µm×5 µm) and representative surface profiles of the materials used in this study for biofilm cultivation **(A)** and the investigation of the bacterial adhesion **(A–D)** are shown in **Fig. 4**. The calculated Rq based on the AFM images (1 µm×1 µm; n = 3, mean ± SD) were 2.2±0.1 nm for the physical vapor deposited titanium thin films, 1.9±0.3 nm for the poly(styrene) surfaces, 16.5±2.1 nm for the ground poly(tetrafluoroethylene) surfaces, and 0.3±0.1 nm for the Borofloat® silicate glass surfaces (**Table 1**). The calculated specific surface area of the materials, skewness, the maximum vertical peak-to-valley distances and the measured contact angles are listed in **Table 1** (n = 3, mean ± SD). The number of peaks and the average distance between the surface peaks on surface line profiles were estimated for characterization of the spatial distribution of the surface features, since the calculated roughness values Rq for TiO_2_ and TCPS were similar. The number of surface peaks of TiO_2_ and TCPS were 24.0±3.2 and 14.8±2.2 per 1 µm, respectively. The average distances between the surface peaks were 42.3±5.5 nm for TiO_2_ and 69.1±10.6 nm for TCPS.

**Table 1:** Surface parameters of the biomaterials and duration and bacterial adhesion rates of the observed adhesion phases.

						Duration [hours]			Adhesion rate [% increase coverage/hour]^b^	
Biomaterial	Rq [nm]^a^	Specific surfacearea [µm^2^]^a^	Skewness	Max vertical peak-to-valley [nm]	Contact angle [degree]	lag-phase I	adhesion II	saturation III	lag-phase I	adhesion II
**TiO_2_**	2.2±0.1	1.037±0.001	0.46±0.11	16.3±1.8	74.3±4.9	<6	6 to 18	>18	0.22	0.70
**TCPS**	1.9±0.3	1.008±0.002	1.13±0.27	15.0±1.5	88.0±1.8	<5	>5	n.o.	0.21	0.51
**PTFE**	16.5±2.1	1.075±0.010	−0.20±0.47	76.27±59.71	113.2±0.8	<3	3 to 10	>10	0.36	2.76
**Glass**	0.3±0.1	1.003±0.0003	0.11±0.13	2.75±0.59	32.7±2.0	<16	>16	n.o.	0.00	0.09

^a^ Values of Rq, specific surface area, skewness and the maximum vertical peak-to-valley distances were calculated based on AFM images with a scan size of 1 µm×1 µm.

^b^ Adhesion rates were calculated for each adhesion phase according to equation (3); n.o. – phase not observed.

Adhesion kinetics of *E. coli* on titanium oxide, tissue culture poly(styrene), poly(tetrafluoroethylene) and Borofloat® silicate glass presented as duration of the observed phases (I–III) in hours and adhesion rates defined as % increase of coverage per hour. For each material, the root mean square roughness Rq, the specific surface area, skewness, the maximum vertical peak-to-valley distance and the measured water contact angle are given (n = 3, mean ± SD).

### Evolution of *Escherichia coli* Biofilms on Titanium Oxide

**Figure 5:**
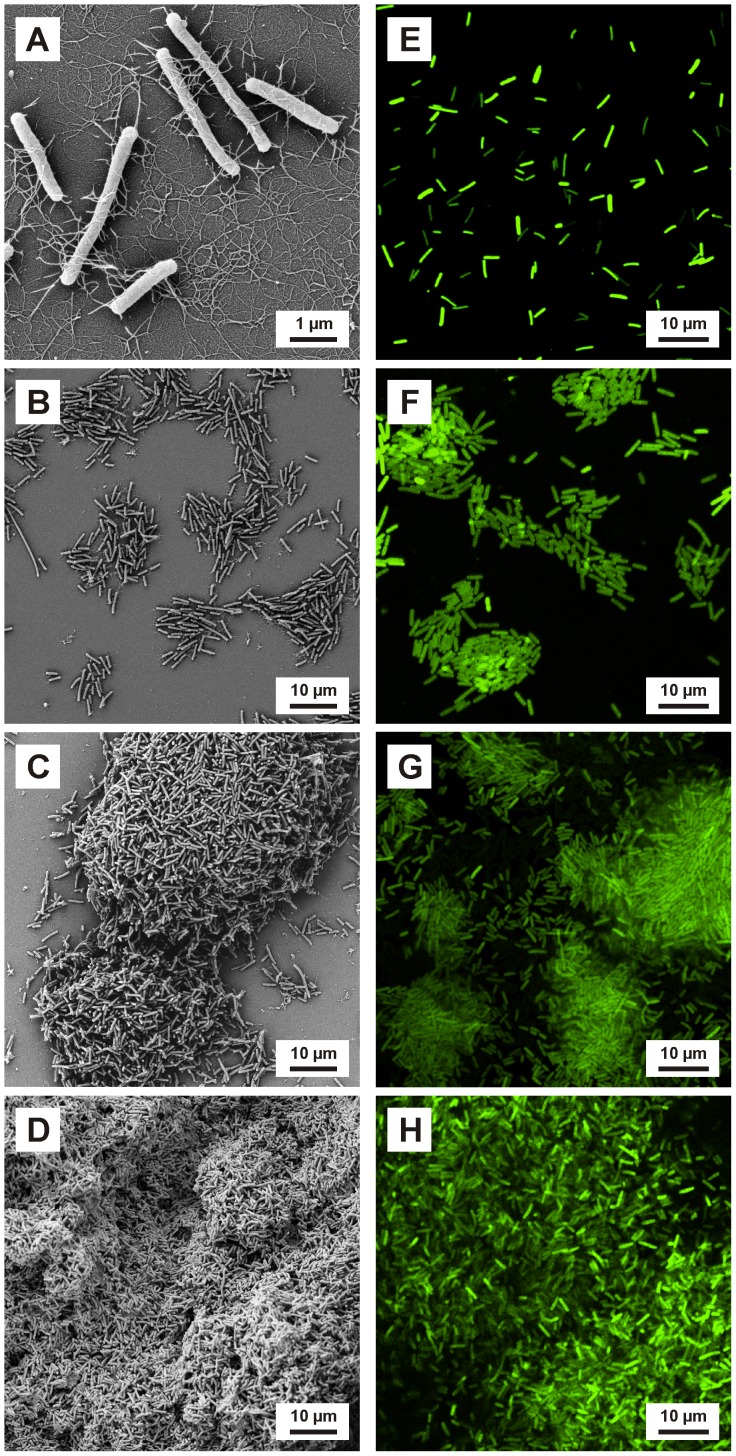
Biofilm formation of *E. coli* on the titanium oxide surface. SEM (**A–D**) and CLSM (**E–H**) images of the biofilm formation of *E. coli* on titanium oxide surfaces cultivated using the nCDFF. The bacteria showed a typical biofilm development including initial adhesion (24 h) (**A, E**), formation of microcolonies (48 h) (**B, F**), early (3 d) (**C, G**) and late maturation (6 d) (**D, H**). Biofilm height and biomaterial surface coverage increased over time.

**Figure 6:**
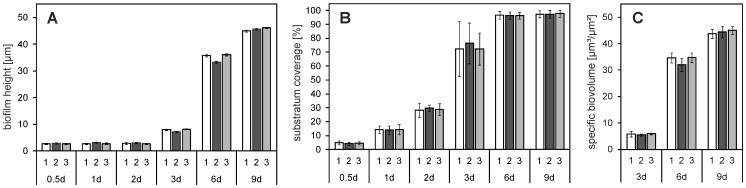
Characterization of *E. coli* biofilms on TiO_2_. Biofilm development of *E. coli* on titanium oxide over time. Biofilms were characterized by height (**A**), substratum coverage (**B**) and specific biovolume (**C**)**.** The statistical analysis (one-way ANOVA) of the results of the three experimental runs (1–3) did not indicate statistically significant differences with p-values >0.05. However, biofilm heights of run 2 at day 3 and 6 differed to that of run 1 and 3 (data not shown).

*E. coli* biofilms were cultivated on TiO_2_ using approach (i) for validating the nCDFF and reproducibility of results. Biofilm formation at 1 d, 2 d, 3 d and 6 d are shown by SEM micrographs **(** **Fig. 5 A–D** **)** and the corresponding CLSM images **(E–H)** (for conciseness, images of 0.5 d and 9 d not shown). Biofilms were characterized by height [µm] **(** **Fig. 6 A** **)**, surface coverage [%] **(B)** and specific biovolume [µm^3^/µm^2^] **(C)** (n = 3 with 5 different points analyzed on each sample, mean ± SD). Biofilm cultivation was performed in triplicate as indicated in **Fig. 6** with the numbers 1–3.

Between 0.5 d and 2 d, the bacteria randomly attached to the surfaces and microcolonies formed. After 1 day extracellular polymeric substances (EPS) were observed **(** **Fig. 5 A** **)**. No increase in biofilm height occurred during the first 2 days (**Fig. 6 A** ****), but an increase in biomaterial surface coverage from 4.5±1.2% to 29.0±3.8% was detected suggesting single cell layer growth until 2 d. With biofilm maturation, the microcolonies increased in size (**Fig. 5 B, C** ****) and height (early maturation). After 6 d and 9 d of growth, TiO_2_ surfaces were nearly completely covered by the biofilm (late maturation) (**Fig. 5 D, H** **;** **Fig. 6 B** ****). Between 6 d and 9 d the average biofilm height increased from 35.1±0.4 µm to 45.5±0.3 µm. Surface coverage was 96.5±2.6% and 97.5±2.6% after 6 d and 9 d, respectively. The specific biovolume [µm^3^/µm^2^] of the biofilms was calculated for 3 d, 6 d and 9 d **(** **Fig. 6 C** **),** given no increase in height occurred during the first 2 days. Between 3 d and 9 d the biovolume increased from 5.7±0.6 µm^3^/µm^2^ to 44.4±1.8 µm^3^/µm^2^.

The statistical analysis of the results (one-way ANOVA) did not indicate statistically significant differences, with p-values >0.05 between the three biofilm cultivation runs for all analyzed parameters. However, biofilm heights of run 2 at day 3 and 6 differed to that of run 1 and 3, but, these variations were small with a difference of run 2 to run 1 and 3 of approximately 0.95 µm (11.7%) at day 3 and 2.7 µm (7.4%) at day 6, respectively.

### *Escherichia coli* Adhesion Kinetics on Biomaterials

**Figure 7:**
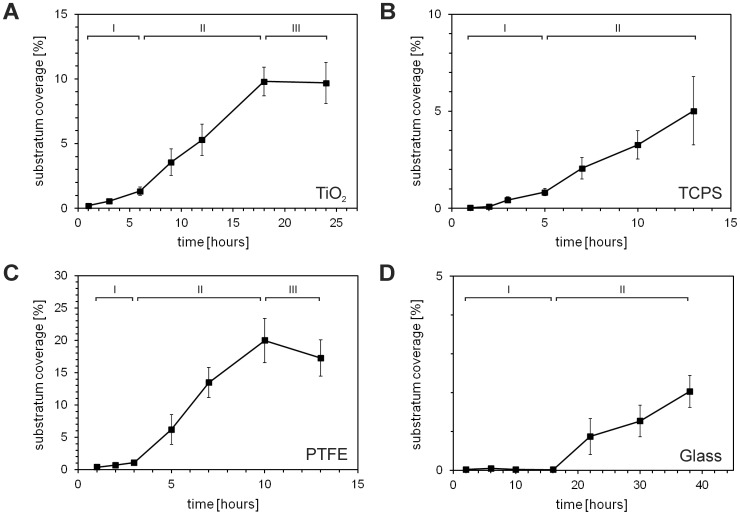
Adhesion kinetics of *E. coli* on different biomaterials surfaces over time. Adhesion kinetics of *E. coli* on different biomaterials surfaces shown as development of substratum coverage over time: titanium oxide (**A**), tissue culture poly(styrene) (**B**), ground poly(tetrafluoroethylene) (**C**) and Borofloat® silicate glass (**D**). The graphs indicate different phases with an initial lag-phase I with no or slow bacterial adhesion, a phase of the fastest, nearly linear adhesion II and for TiO_2_ and PTFE a phase of saturation III with no further increase in surface coverage.

Bacterial adhesion was investigated on four different biomaterials surfaces using the nCDFF in combination with a continuous culture of *E. coli* based on approach (ii) **(** **Fig. 1** **)**. The development of the biomaterials surface coverage by *E. coli* over time is shown in **Fig. 7** for TiO_2_ **(A)**, TCPS **(B)**, PTFE **(C)** and glass **(D)** (n = 3, with 5 different points analyzed for each sample, mean ± SD). The graphs indicate different phases during the adhesion process. In particular, an initial lag-phase I with no or slow bacterial adhesion, a phase of fastest adhesion II and for TiO_2_ and PTFE a phase of saturation III with no further adhesion were observed.

Assuming the bacterial adhesion in each phase is linear over time, the adhesion rates r for the initial lag-phase I and fast adhesion phase II were, thus, calculated by equation (3):
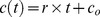
(3)with c the coverage, c (t) the coverage over time t, and c_0_– coverage at the beginning of the phase. The time range and the adhesion rates r, representing the average increase of coverage in % per hour, are given for phase I and II and each material in **Table 1**.

On TiO_2_ and the TCPS surfaces, similar adhesion rates were observed with lengths of the lag-phases I of 6 h and 5 h, respectively, and an adhesion rate during the fast adhesion phase II of 0.70% and 0.51%. On the PTFE surfaces, the bacteria showed the fastest adhesion accompanied by the shortest lag-phase. An increase of the substratum coverage of 0.36% and 2.76% per h during the lag-phase and the fast adhesion phase, respectively, was measured. The slowest adhesion was observed on the glass surfaces with a lag-phase of 16 h and a fast adhesion phase with an increase of coverage of 0.09% per h. The adhesion rates were significantly increased in the fast adhesion phase compared to the lag-phase. No further increase in material surface coverage over time was observed for PTFE after 10 h and for TiO_2_ after 18 h.

On the TCPS surfaces, the point of transition from lag-phase I to adhesion phase II is not as clear as for the other three materials. The time point was set to t = 5 h, since at this point a noticeable increase of the adhesion rate occurred. Alternatively, t = 2 h would have been a possible point to set as the end of the lag-phase I and the beginning of phase II. In this case, the rate of adhesion in the lag-phase I is r = 0.05%/h and in the adhesion phase II r = 0.45%/h. Nevertheless, using these alternative values does not change the outcome and main conclusion of the study.

**Figure 8:**
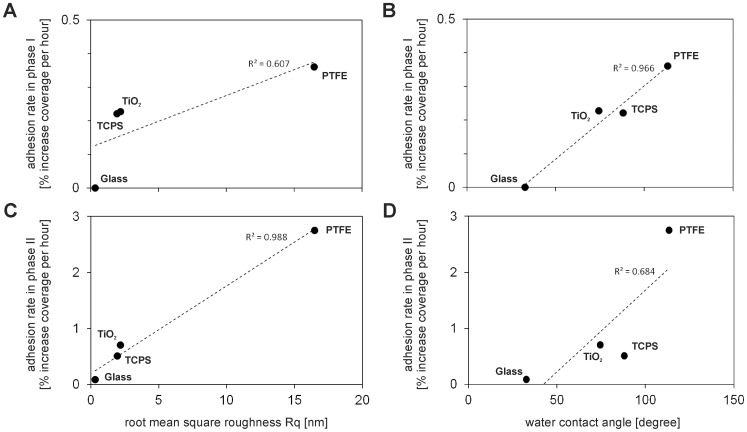
Correlation of bacterial adhesion kinetics with biomaterials surface nanoroughness and hydrophobicity. Adhesion rates of *E. coli* in phases I and II on titanium oxide, tissue culture poly(styrene), ground poly(tetrafluoroethylene) and Borofloat® silicate glass in dependence of root mean square roughness (**A, C**) and in dependence of water contact angle (**B, D**) of the biomaterials. The linear regression and the coefficient of determination R^2^ are given.

With increasing surface nanoroughness of the biomaterials (Rq = 0.3 nm to 16.5 nm), the lag-phases became shorter (from 16 hours to 3 hours) **(** **Table 1** **)**. As nanoroughness increases, the adhesion rates of lag-phase I and adhesion phase II **(** **Fig. 8 A, C** **)** increased. A linear regression analysis was applied to identify which parameter is more directly correlated with the bacterial adhesion rates [9]. The correlation of adhesion rates of the lag-phase I with surface roughness yields a correlation coefficient R^2^ = 0.61 for a linear regression. The correlation of adhesion rates during the fast adhesion phase II with roughness was clearly stronger and almost linear with R^2^ = 0.99.

As water contact angle increases, the lag-phases I became shorter **(** **Table 1** **)** and the adhesion rates of the lag-phase I increased (R^2^ = 0.97 for a linear regression) **(** **Fig. 8 B** **)**. The bacterial adhesion rates on glass, TiO_2_ and PTFE of adhesion phase II also increased with increasing water contact angle **(** **Fig. 8 D** **)**. Nevertheless, the adhesion rate in phase II on the TCPS surfaces does not correlate that well. The linear regression analysis revealed a correlation coefficient of R^2^ = 0.68. Thus, the correlation of the adhesion rates with the surface contact angles during phase II is not as clear as the correlation with the surface nanoroughnesses.

## Discussion

### Non-constant-depth Film Fermenter for Biofilm Cultivation and Investigation of Microbial Adhesion

In this study, the CDFF was modified for the cultivation of non-z-restricted biofilms (non-constant-depth film fermenter, nCDFF) **(** **Fig. 2** **)** to meet the requirements for investigating biofilm formation at low shear conditions, with non-unidirectional fluid flow and with constant conditions throughout the biofilm reactor. Using the nCDFF and chemostat-grown *E. coli* as inoculum, biofilms were cultivated on TiO_2_ and tested for reproducibility.

The specific design of the nCDFF (based on the CDFF) enables a cultivation of biofilms at low shear conditions. Due to the small volume of the wells, no stirring is necessary for mixing the fluid. Depending on the feeding rate, the total volume is replaced within minutes (see **Fig. 1** **,** **2**). The permanent exchange of the inoculum/growth medium prevents the accumulation of metabolic waste products, signaling molecules and dead cells. Hence, conditions for bacterial adhesion and subsequent biofilm formation are constant throughout all sample wells of the nCDFF. The nCDFF, thus, fulfills the requirements necessary for adequate testing of biomaterials, i.e. implants, which are used at locations in the human body with low shear conditions and non-unidirectional fluid flow.

Oral bacteria occur in their natural habitat, the oral cavity, in a commensal community of several species. Thus, using multispecies continuous cultures of oral bacteria for biofilm (plaque) cultivation in the CDFF is obvious. However, significant variations in species composition between different runs of the system had been observed despite the multispecies continuous culture having reached a quasi steady state [19]. These variations might be due to spontaneous mutations taking place continuously. Furthermore, natural saliva or plaque used for inoculation of the chemostat culture is variable and not defined in its species composition. Results of the current study show that using a single-species continuous culture prevents significant variations between the experimental runs **(** **Fig. 6** **)**. Typical biofilm development was observed as described in literature [45] including initial bacterial adhesion, microcolony formation, growth of the colonies and maturation of the biofilm **(** **Fig. 5** **)**. To investigate, e.g. co-adhesion, competitive behavior or mixed biofilm formation, it is intended to use several single-species continuous cultures in parallel. This is expected to allow for exact definition of species composition and the quantity of each species and is, therefore, highly replicable.

The nCDFF in combination with a chemostat is suitable for both biofilm studies as well as investigating bacterial adhesion, because parallel but asynchronous growth processes are avoided. Due to steady state conditions within the chemostat, the amount of available carbon source, e.g. glucose, (if used as growth limiting factor) is below the detection limit. Any remnants of glucose are consumed during the passage of the bacterial suspension through the silicon tubes to the nCDFF for inoculation. Thus, no glucose is available in the nCDFF as shown in this study. Furthermore, the bacterial suspension in the wells is constantly replaced. Growth within the bacterial suspension in the sample wells can, thus, be excluded. The saturation phases observed in this study during adhesion of *E. coli* on TiO_2_ and PTFE **(** **Fig. 7 A, C** **)** indicated that no growth of bacteria took place once adhered to the surfaces. In this phase, no increase of surface coverage was observed suggesting that no further bacterial adhesion had occurred either. The physiologically intact chemostat-grown cells can, thus, be directly used to investigate adhesion within the nCDFF. No previous washing, centrifugation and resuspension steps are necessary and, thus, adhesion of bacterial cells won’t be affected.

Biofilm cultivation and adhesion test were performed at room temperature. Microbial growth rate and, thus, the time range for biofilm formation, are temperature-dependent. Yet, the dynamics of biofilm development nor the reproducibility of the cultivated biofilms are not affected. Thus, reproducing the physiological conditions found in the human body with temperatures of 37°C is not essential for investigating the influence of biomaterials surface properties on the microbial adhesion and biofilm formation. At constant cultivation conditions, differences in the microbial adhesion can be clearly attributed to variations in the biomaterials surface properties.

Different methods for sample preparation should be discussed for the introduced *in vitro* test system for biofilm cultivation and for investigation of bacterial adhesion. Biofilm samples can be investigated microscopically (e.g. with CLSM) in both a fully hydrated and completely dried state. Characterizing adhered cells and fully hydrated biofilms enables a realistic definition of the 3D structures. Fixation and drying steps may change the localization of bacteria on the surfaces and may affect the structure of the biofilms. Nevertheless, the error introduced by this effect should have been small and consistent for all materials, since all samples in this study were handled equally during preparation and with special care. Thus, the observed differences of adhesion on the different materials are unlikely to be artifacts of preparation, but, real differences in the adhesion of the bacteria. For testing the reproducibility of the cultivated biofilms, the protocol used for sample preparation did not affect the results. Advantages of the used protocol are that embedding the samples protects them against fading of the fluorescence during microscopy due to exposure to (laser) light, and that the samples can be stored for months. Furthermore, an immediate fixation of the samples allows for high sampling throughput because the state of the samples at the point of sampling is preserved. This is of considerable importance for high temporal-resolved sampling and sampling time points close to each other.

In summary, the nCDFF introduced here is a suitable system and valuable tool for cultivating non-z-restricted biofilms and for investigating bacterial adhesion at constant and low shear conditions. Using a chemostat culture for inoculating the nCDFF significantly enhanced the reproducibility of the cultivated biofilms. Furthermore, bacterial adhesion can be investigated with viable cells and without asynchronous, competing growth processes. This system, thus, considerably adds to and enhances available *in vitro* testing systems for bacterial adhesion and biofilm formation.

The nCDFF in combination with a single-species continuous culture enables reproducible analysis of biomaterials surface properties correlation with bacterial adhesion and biofilm formation at defined and constant experimental conditions. It is therefore a crucial prerequisite to develop and validate modifications and treatments of biomaterials surfaces with the aim to prevent microbial adhesion and, consequently, the development of biomaterial-associated infections.

### *Escherichia coli* Adhesion Kinetics and Impact of Biomaterials Surface Nanoroughness and Hydrophobicity

Investigating microbial adhesion on biomaterials over time (adhesion kinetics) and in context of materials surface properties, leads towards identifying mechanisms fundamentally involved in the development of BAIs. Biomaterials used as implants in the human body differ in their physical as well as their chemical surface properties. So, multi-factorial investigations are necessary to evaluate the composite impact of these factors on microbial adhesion, and to subsequently inform the development of suitable biomaterials surface modifications preventing BAIs.

The nCDFF was used to examine the adhesion of *E. coli* on four different biomaterials over time and with respect to the materials surface nanoroughness and hydrophobicity. The biomaterials used here represent a broad range of materials with significantly different physico-chemical surface properties. Thus, more general conclusion can be drawn. Furthermore, using a single-species chemostat-culture, providing intact bacterial cells, ensured valid results for the adhesion study.

On materials surfaces exposed to, e.g., growth medium, bacterial culture or human body fluid, a conditioning film is formed [46], [47], where firstly small molecules (mainly water and ions) adsorb to the surface within seconds and small organic molecules, proteins and polysaccharides start to cover the surface within the next minutes. However, the accumulation of the conditioning film continues for hours after first exposure [47], [48]. The formation of a conditioning film on the biomaterials surfaces during the lag-phase is most likely [48]. Proteins tend to adsorb to a greater extent to hydrophobic surfaces than to hydrophilic surfaces [49] which, in turn, could have led to a faster conditioning film formation on more hydrophobic surfaces. Depending on the composition of the conditioning film and the species, bacterial adhesion is promoted or inhibited [8], [47]. Nevertheless, the results indicate that a conditioning film has probably promoted bacterial adhesion, since the durations of the lag-phases decreased and the adhesion rates increased with increasing materials surface hydrophobicity **(** **Table 1** **)**.

However, the durations of the lag-phases have been assessed based on the adhesion curves. This is a rough estimation, since the precision depends on the sampling frequency. Nevertheless, this has not affected the general correlation found between the duration of the lag-phase and the materials surface hydrophobicity.

Bacterial adhesion already occurred during the lag-phase I, although, the adhesion rates were lower compared to the rates in phase II **(** **Fig. 7** **)**. Bacteria may adhere to surfaces without a conditioning film [8]. Gram-negative bacteria, such as *E. coli*, with moderately hydrophobic to hydrophobic cell surfaces preferentially attach to more hydrophobic surfaces [36]–[38], [50]. Hence, with increasing biomaterials surface hydrophobicity, increased bacterial adhesion during the lag-phase was observed here, presumably in parallel to the formation of a conditioning film. This, in turn, could also have led to the shorter lag-phases seen here.

The adhesion rates of *E. coli* during phase II significantly increased with increasing surface roughness **(** **Fig. 8 A** **)**. These results are consistent, e.g., with the findings of currently published studies. Singh *et al.* [51] described a significantly higher adhesion of *E. coli* and *Pseudomonas aeruginosa* on supersonic cluster beam deposited titanium thin films with increasing surface nanoroughness from 16.2 nm to 21.7 nm. Webb *et al.* [52] found the extent of *Staphylococcus aureus* cell attachment to be greater on sub-nanometrically smooth surfaces with higher average and root mean square roughness. However, the underlying mechanisms have not been clearly identified in these studies. It was suggested that greater adhesion of the bacteria to the rougher surfaces may be due to their greater specific surface area. The contact between the bacterial cell and the materials surface is mediated mainly by nanoscaled cell surface structures, such as pili, fimbrae and curli. Surfaces with a higher roughness also in the nanometer range provide more area available for contact between cell and materials surface.

The root mean square roughness used in this study to describe the biomaterials surface topography is a one dimensional amplitude parameter. Rq describes the typical height of the peaks on the surface and gives no indication of their spatial distribution. The roughness values calculated for the TiO_2_ surfaces and the TCPS are in the same range **(** **Table 1** **)**. Nevertheless, as shown in the surface profiles **(** **Fig. 4** **)**, the topography of these two materials is clearly different. The number of topographical surface peaks was almost two times higher on the TiO_2_ surfaces compared to the TCPS surfaces. Accordingly, the average distance between the peaks was nearly two times greater on the TCPS surfaces compared to the TiO_2_ surfaces. Thus, also the specific surface area of the TiO_2_ was higher compared to that of TCPS. The higher specific surface area of TiO_2_ may be an explanation for the slightly increased adhesion rate of the bacteria in phase II to the TiO_2_ surfaces compared to the TCPS surfaces (see **Fig. 8 C**). Siegismund *et al.* [53] showed based on mathematical modeling that in addition to amplitude roughness parameters, such as Rq, also the surface peak density as spacing surface parameter distinctively influences the interaction energies between microbial cell and materials surface. With increasing peak density, the interaction energy increases. The higher adhesion rates of *E. coli* observed on TiO_2,_ thus, may be due to the higher number of the materials surface peaks.

Skewness as an additional roughness parameter is suitable for a more precise description of surface nanoarchitecture with regard to horizontal dimension [54]–[55]. The skewness significantly differed between the TiO_2_ and the TCPS surfaces **(** **Table 1** **)** with 0.46±0.11 and 0.13±0.27, respectively, which emphasizes the different surface topographies of both materials. Positive skewness indicates that the bulk of the materials is below the surface profile mean line and corresponds to the presence of more occasional deep valleys and less high peaks. Thus, on the TiO_2_ more cavities for anchoring of the bacterial cells were available which, in turn, may also explain the higher surface coverage and higher adhesion rates of the bacteria on the TiO_2_ surfaces compared to TCPS.

In contrast to the results obtained in the current study, the authors found previously [31] that the surface coverage of titanium thin films by *E. coli* was reduced with increasing surface nanoroughness from 2.0 nm to 6.1 nm. Mitik-Dineva *et al.* [56] investigated bacterial adhesion on as-received and chemically etched glass surfaces. Etching resulted in a 70% reduced surface nanoroughness and an increase of the number of adhered bacteria by a factor of three. However, chemical modifications due to the etching were not completely discussed. The results presented in this study and the partially conflicting results reported in literature clearly show that further analysis is required to identify fundamental mechanisms of how surface roughness on a nanometer scale affects bacterial adhesion. This study provided the necessary tools.

The contact angle, hence, hydrophobicity of materials surfaces depends on the surface roughness [57]–[58]. Nevertheless, this effect is small for variations of roughness in the low nanometer range. For example, Cai *et al.* [59] showed that the contact angles of physical vapor deposited titanium thin films with Rq between 1.9 nm and 20.7 nm were not significantly different. The contact angles of the biomaterials used in this study are, thus, mainly determined by the material itself and rather by the different surface roughnesses.

In general, the influence of materials surface hydrophobicity on bacterial adhesion is strongly species-dependent [36]–[38]. Adhesion forces between Gram-negative bacteria, such as *E. coli*, *P. aeruginosa* and *P. stutzeri*, and materials surfaces are higher for more hydrophobic surfaces. *Vice versa*, the adhesion forces are higher between Gram-positive bacteria, such as *S. aureus* and *S. epidermidis*, and hydrophilic surfaces. The adhesion rates of *E. coli* observed in this study increased with increasing surface hydrophobicity as expected **(** **Fig. 8 B, D** **)**.

The correlation of bacterial adhesion rates and biomaterials surface properties and a linear regression analysis can give an indication which surface factor affects adhesion to a greater extent [9]. The bacterial adhesion rates during lag-phase I were stronger correlated with the surface contact angles than the adhesion rates during phase II (see **Fig. 8 B, D**). The opposite was seen for the biomaterials surface nanoroughness. The adhesion rates during the lag-phase I were less clearly correlated with the surface roughness than the adhesion rates during phase II (see **Fig. 8 A, C**). Based on these observations, a stronger impact of materials surface hydrophobicity on the bacterial adhesion during the initial lag-phase I and a stronger impact of surface nanoroughness on bacterial adhesion during the fast adhesion phase II can be assumed.

The formation of a conditioning film masks the impact of surface hydrophobicity to some extent. Bruinsma *et al.* [8] showed that the surface contact angle of hydrophobic hydrogel contact lenses was reduced from 106° to 69° due to the formation of a conditioning film. Bakker *et al.* [9] demonstrated that the contact angle of hydrophilic polyurethane coatings increased by 8° due to conditioning film formation. It can, therefore, be hypothesized that the formation of a conditioning film during lag-phase increased the contact angle of glass (∼32°) and reduced that of PTFE (∼113°). Hence, the stronger influence of surface roughness during phase II can be explained by a probably reduced influence of hydrophobicity. To test this hypothesis, conditioning film formation caused by the bacterial culture and the effects on the biomaterials surface properties will be addressed in further studies.

Bacterial adhesion and subsequent biofilm formation is a time-dependent process. Investigating bacterial adhesion over time thus provides much needed insight into bacterial adhesion in response to the materials surface properties. This study provides the insight by means of bacterial adhesion kinetics on different biomaterials. It demonstrates that bacterial adhesion is governed by an interplay of different physico-chemical surface properties of the biomaterials. Nevertheless, the impact of one factor may exceed the influence of another depending on the phase of bacterial adhesion. The very early adhesion of *E. coli* was found to be remarkably reduced with increasing hydrophilicity. Thus, hydrophobicity-reducing treatments of biomaterials surfaces such as coatings, UV- or oxygen plasma treatments may reduce adhesion and adhesion rates of *E. coli*. A combination of these modifications with, e.g., surface structuring to promote human tissue cell integration is, therefore, one promising approach for preventing BAIs most commonly associated with *E. coli*.

## Summary and Conclusions

This study introduced the nCDFF for cultivating non-z-restricted biofilms under constant and low shear conditions. Inoculation of the nCDFF with a single-species continuous culture minimized variations between different experimental runs and enhances reproducibility of the cultivated biofilms. The modification of the previously described CDFF system, substantially broadens its applicability in biomaterials science.

The nCDFF inoculated with a single-species continuous culture in particular allows the adequate examination of microbial adhesion. The continuous culture provides intact bacterial cells that were not previously weakened by centrifugation and/or resuspension steps. Moreover, bacterial growth during adhesion is minimized. Results of adhesion studies using this system are, thus, reliable and valid. The nCDFF considerably expands the pool of available *in vitro* testing systems for bacterial adhesion and biofilm formation. This system is an invaluable tool in the analysis of modifications and treatments of biomaterials surfaces to prevent microbial adhesion and the subsequent development of BAIs.

This study provides detailed insight in the adhesion of *Escherichia coli* on various biomaterials over time and depending on the materials nanoroughness and hydrophobicity. Bacterial adhesion kinetics included an initial lag-phase I, a fast adhesion phase II and a phase of saturation III. Hydrophobicity was found to mainly affect the very early bacterial adhesion. A combination of biomaterials surface modifications to both increase surface hydrophilicity and promote tissue cell integration (e.g., by surface structuring) is a promising and informed approach for preventing BAIs associated with *E. coli*.

## Supporting Information

Figure S1 **Structure of plasmid pMK3c2GFPuv.** ColE1 ori - origin of replication, *lac*I promoter - promoter of *lac*I repressor gene, *lac* promoter - promoter of *lac* operon gene, GFPuv - gene of improved GFP variant, t_LPP - transcription terminator of LPP gene, sok-RNA - antisense RNA blocking hok-mRNA (suppressor of killing), *hok* - gene encoding host killing protein, t-hok - transcription terminator of hok gene, *aph*A1 - gene of aminoglycoside-phosphotransferase conferring kanamycin resistance. The *lac*I gene in the intermediate plasmid pMK31GFPuv was inactivated by excision of the large *Psp*1406I/*Nar*I DNA fragment encoding the amino acids (aa) 6 to 331 of the LacI repressor. The tandem SD sequence in front of the GFPuv gene is structured as shown in the box.
(TIF)

Figure S2 **Flow chart of the two approaches used in this study and opportunities for modifications.** Biomaterials are inoculated for a certain period of time to cultivating non-z-restricted biofilms using the nCDFF. Then sterile culture medium is supplied to maintain biofilm growth. For bacterial adhesion kinetics analysis, biomaterials are permanently inoculated. Different pathogen species can be cultivated in a chemostat and used for inoculation of the nCDFF. The nCDFF can be inoculated with one continuous culture or several in parallel. Various methods are available for characterization of the adhered bacteria and the cultivated biofilms, respectively. Here suitable samples for microscopy and subsequent image analysis are indicated.
(TIF)

Text S1 **Supplementary methods.** Construction of plasmid pMK3c2GFPuv.
(DOC)
