# Supplementary figures and images for: Reproducible Biofilm Cultivation of Chemostat-Grown Escherichia coli and Investigation of Bacterial Adhesion on Biomaterials Using a Non-Constant-Depth Film Fermenter
Source: PLoS One. 2014 Jan 3;9(1):e84837. doi: 10.1371/journal.pone.0084837 (PMC3880331; doi:10.1371/journal.pone.0084837)

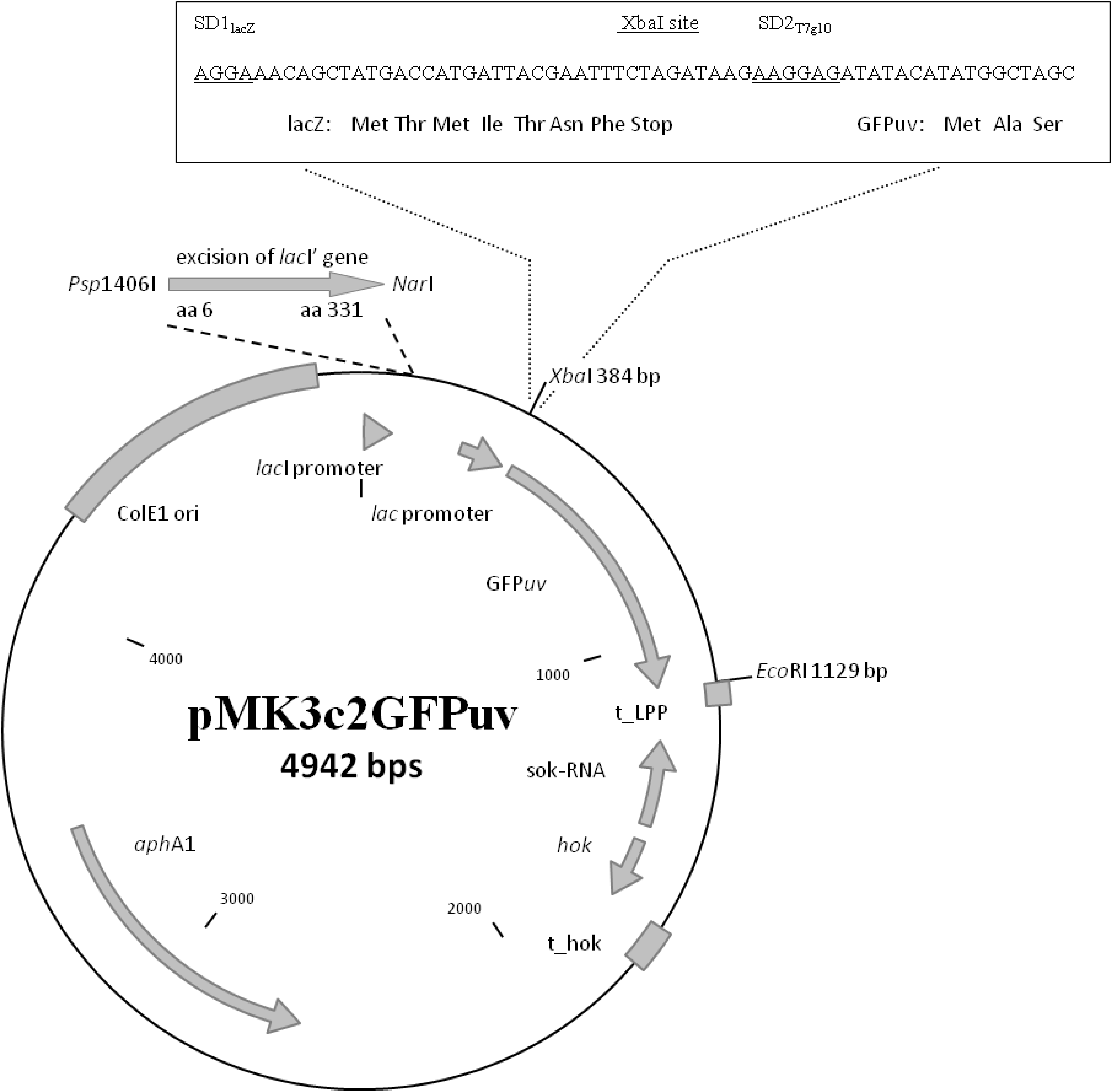

Supplement: Figure S1 — Structure of plasmid pMK3c2GFPuv. ColE1 ori - origin of replication, lacI promoter - promoter of lacI repressor gene, lac promoter - promoter of lac operon gene, GFPuv - gene of improved GFP variant, t_LPP - transcription terminator of LPP gene, sok-RNA - antisense RNA blocking hok-mRNA (suppressor of killing), hok - gene encoding host killing protein, t-hok - transcription terminator of hok gene, aphA1 - gene of aminoglycoside-phosphotransferase conferring kanamycin resistance. The lacI gene in the intermediate plasmid pMK31GFPuv was inactivated by excision of the large Psp1406I/NarI DNA fragment encoding the amino acids (aa) 6 to 331 of the LacI repressor. The tandem SD sequence in front of the GFPuv gene is structured as shown in the box. (TIF) [file pone.0084837.s001.tif]

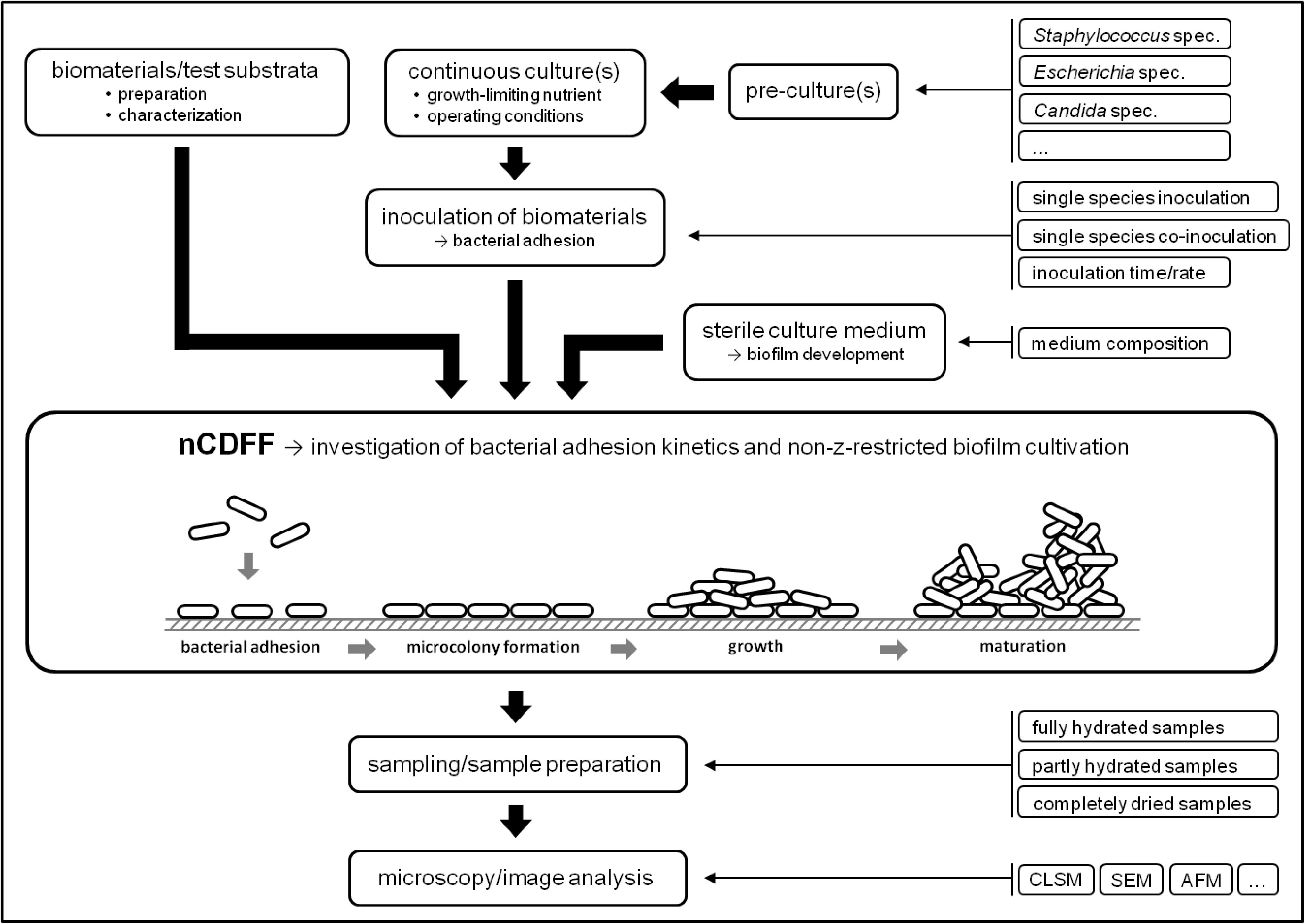

Supplement: Figure S2 — Flow chart of the two approaches used in this study and opportunities for modifications. Biomaterials are inoculated for a certain period of time to cultivating non-z-restricted biofilms using the nCDFF. Then sterile culture medium is supplied to maintain biofilm growth. For bacterial adhesion kinetics analysis, biomaterials are permanently inoculated. Different pathogen species can be cultivated in a chemostat and used for inoculation of the nCDFF. The nCDFF can be inoculated with one continuous culture or several in parallel. Various methods are available for characterization of the adhered bacteria and the cultivated biofilms, respectively. Here suitable samples for microscopy and subsequent image analysis are indicated. (TIF) [file pone.0084837.s002.tif]
