# Supplementary material for: Reproducible Biofilm Cultivation of Chemostat-Grown Escherichia coli and Investigation of Bacterial Adhesion on Biomaterials Using a Non-Constant-Depth Film Fermenter
Source: PLoS One. 2014 Jan 3;9(1):e84837. doi: 10.1371/journal.pone.0084837 (PMC3880331; doi:10.1371/journal.pone.0084837)
Supplement: Text S1 — Supplementary methods. Construction of plasmid pMK3c2GFPuv. (DOC) [file pone.0084837.s003.doc]

**Supplement**

**Construction of plasmid pMK3c2GFPuv**

Starting with plasmid pACK02scKan-hs [1], a series of expression vectors with modular elements was constructed. One derivative, pMK31scFvDhlx, contains the *lac*I repressor gene and the *lac* promoter (controlling expression of the recombinant product gene), the genes of the *hok/sok* system (enhancing plasmid stability by post-segregational killing of plasmid-free cells) [2-3], the *aph*A1 gene (conferring kanamycin resistance), and the ColE1 origin of replication. A tandem ribosome binding site (including a Shine-Dalgarno (SD) sequence,a short *lac*Z coding sequence, followed by an *Xba*I restriction site and the second strong SD sequence [T7g10]) is localized between the *lac* promoter and the start codon of the recombinant product gene. This tandem SD sequence had been described in detail for another derivative of this vector series, pHKK [4].

Plasmid pMK31scFvDhlx (6.183 bp) was digested using endonucleases *Xba*I and *EcoR*I to excise the gene of the recombinant product scFvDhlx and the 5’ flanking SD sequence T7g10. The 5.184 bp backbone fragment was purified following gel electrophoresis using a gel extraction kit.

The GFPuv encoding gene was amplified using PCR and plasmid pBAD-GFPuv [5] as template. The 5’ flanking primer includes an *Xba*I restriction site as well as the identical SD sequence variant SD2 T7g10 of pBAD-GFPuv. The 3’ flanking primer contains the sequence encoding the GFPuv C-terminus and an *EcoR*I restriction site. The PCR product was also digested with endonucleases *Xba*I and *EcoR*I, and the resulting truncated PCR fragment (745 bp) was purified using a nucleotide removal kit. Ligating the *Xba*I/*Eco*RI fragment of plasmid pMK31scFvDhlx and subsequently selecting for green fluorescent transformants using LB ager plates with 0.1 mM IPTG resulted in amplifying and validating the intermediate plasmid pMK31GFPuv.

Finally, the *lac*I gene in pMK31GFPuv was almost completely deleted by restriction with singular cutters *Psp*1406I and *Nar*I inside the *lac*I gene, and by religating the compatible ends without the 978 bp *lac*I fragment. The structure of the resulting plasmid pMK3c2GFPuv is shown in **Fig. S1**.

Constitutive GFPuv expression is controlled by both *lac*I and *lac* promoter. This promoter tandem and the SD tandem allow a strong expression of GFPuv. The hok/sok suicide system minimizes segregational plasmid loss and, thus, ensures a homogeneous fluorescent cell population.

**References**

[1] Kujau MJ, Riesenberg D (1999) Co-operative effects of protein engineering and vector optimization on high yield expression of functional bivalent miniantibodies in *Escherichia coli*. Microbiol Res 154: 27-34.

[2] Gerdes K (1988) The parB (*hok/sok*) locus of plasmid R1: a general purpose plasmid stabilization system. Biotechnology 6: 1402-1405.

[3] Thisted T, Nielson AK, Gerdes K (1994) Mechanism of post-segregational killing: translation of Hok, SrnB and Pnd mRNAs of plasmid R1, F and R483 is activated by 3’-end processing. EMBO J 13: 1950-1959.

[4] Horn U, Strittmatter W, Krebber A, Knüpfer U, Kujau MJ, et al. (1996) High volumetric yields of functional dimeric miniantibodies in *Escherichia coli*, using an optimized expression vector and high-cell-density fermentation under non-limited growth conditions. Appl Microbiol Biot 46: 524-532.

[5] Crameri A, Whitehorn EA, Tate E, Stemmer WPC (1996) Improved green fluorescent protein by molecular evolution using DNA shuffling. Nat Biotechnol 14: 315-319.
